# Current Density Optimization for SERS Substrates Using Galvanostatically Anodized TiO_2_ Nanostructures Decorated with Pulsed-Electrodeposited Silver Dendrites

**DOI:** 10.3390/ma18184366

**Published:** 2025-09-18

**Authors:** Marcos Luna-Cervantes, Josué Ismael García-Ramírez, Julián Hernández-Torres, Luis Zamora-Peredo

**Affiliations:** Centro de Investigación en Micro y Nanotecnología, Universidad Veracruzana, Boca del Río 94294, PC, Mexico; zs21023454@estudiantes.uv.mx (J.I.G.-R.); julihernandez@uv.mx (J.H.-T.)

**Keywords:** constant current anodization, nanotubes, nanograss, nanoparticles, dendrites, SERS

## Abstract

In this work, the effect of the applied current density (J) during galvanostatic anodization of titanium (5–30 mA/cm^2^) on the TiO_2_ morphology and its influence on the SERS performance after pulsed silver electrodeposition (5 mA/cm^2^, 400 cycles, 50 ms ON/250 ms OFF) was evaluated. SEM micrographs showed a transition from nanotubes with scarce nanograss to a dense coverage of nanograss over the surface, which conditioned the formation and distribution of silver nanoparticles and dendrites. Using methylene blue as a probe molecule, the substrate anodized at 15 mA/cm^2^ exhibited the highest SERS intensity, attributed to the high density of hot spots vertically distributed in the 3D dendrites. The optimized substrate demonstrated detection of methylene blue down to 1 × 10^−11^ M and reached an analytical enhancement factor of 7 × 10^7^, highlighting its strong performance. These results establish galvanostatic anodization as a novel and effective route for designing reproducible and scalable TiO_2_/Ag substrates with high SERS response.

## 1. Introduction

For decades, the study and fabrication of devices based on titanium dioxide nanostructures (TNSs) have attracted great interest for diverse applications, including perovskite solar cells [1], photocatalysis [2], environmental remediation [3], supercapacitors [4], and analyte detection using the Surface-Enhanced Raman Scattering (SERS) technique [5]. The outstanding performance of these devices has driven this particular interest due to the excellent electronic and optical properties of TiO_2_ [6,7]. Anodization is one of the most widely used methods to fabricate TNSs, due to its low cost, reproducibility, morphological control, and scalability [8]. Several studies have been published regarding this electrochemical technique, including the parameters involved and their impact on the resulting morphology [9]. In terms of technical implementation, anodization can be performed under constant voltage (CVA), also known as potentiostatic anodization [10], pulsed voltage (PVA) [11], or constant current (CCA) [12] regimes. To date, there are few studies on the CCA methodology applied to TiO_2_.

It is well known that in CVA, the applied voltage controls the tube diameter; however, their length is strongly determined by the synthesis time, due to the exponential decrease in current density (J), which is a key factor for this parameter [13,14,15]. This decrease in J is influenced by the appearance and growth of the initial oxide layer (barrier layer) [16]. In contrast, in CCA, the growth rate of the nanostructures is directly associated with the applied current density [17]. Among the most common electrolytes used are those based on monoethylene glycol (MEG) and ammonium fluoride (NH_4_F) [18]. The effects of NH_4_F concentration, volume ratio, or water content have also been the subject of several studies under CVA conditions [19,20,21]. Depending on the NH_4_F concentration, there is a critical voltage above which the TiO_2_ growth–dissolution rate is no longer in equilibrium, making it impossible to determine the thickness or structure obtained accurately [22]. In addition, the electrolyte tends to heat up, which reduces the viscosity of MEG and, in turn, increases the conductivity of the medium. As a result, the current density increases significantly, leading to electropolishing that dissolves all the previously formed TiO_2_, or even cutting the titanium substrate exactly at the electrolyte–air interface [23,24]. These limitations can be mitigated by implementing a cooling system, adding additives to prevent dielectric breakdown, or reducing the NH_4_F concentration [25,26]. Galvanostatic anodization, also known as constant current anodization (CCA), provides a practical solution to overcome these issues. Under CCA, the voltage reaches a limit according to the system conditions (initial temperature, NH_4_F concentration, current density) and remains nearly constant throughout the process. This stability reduces the rapid and uncontrolled increase in temperature. The advantage of CCA is that the TNS is obtained in a lower time [27]. In addition, the oxide thickness exhibits a linear growth behavior, enabling straightforward thickness estimation.

Galvanostatic anodization is not limited to titanium; similar approaches have been successfully applied to other valve metals such as Zr, Nb, and Ta to produce self-organized oxide layers with tubular or porous morphologies [28,29,30,31]. These materials have been mainly explored in photocatalysis, energy storage, and biomedical applications, but in principle, they could also serve as platforms for SERS-active substrates. However, reproducibility in SERS performance may be more challenging due to differences in oxide chemistry, dielectric constants, and structural stability, which influence nanoparticle anchoring, hot spot formation, and plasmonic coupling. Thus, while our study focuses on TiO_2_, the galvanostatic anodization strategy highlights a framework that could be extended to other valve metals, provided these challenges are carefully addressed.

As mentioned, one of the applications of TNSs is in the SERS field [32]. This technique has been explored and widely used for the detection of biological [33] or chemical analytes [34] due to its high sensitivity. Under optimal conditions, it has even been possible to detect a single analyte molecule [35,36,37,38]. This remarkable sensitivity is due to the localized surface plasmon resonance effect (LSPR). TNS substrates have commonly been functionalized with silver nanostructures (AgNSs) to be used in these applications. The morphological characteristics of the substrates significantly influence how the AgNSs adhere and thus modify the SERS response. AgNSs can be fabricated in various geometries, including nanorods, nanosheets, pyramids, flower-like particles, and dendrites [39,40,41]. Among these, hierarchical fractal dendrites (AgDs) stand out for their remarkable electronic and optical properties [42]. This outstanding performance is attributed to their large surface area, which is derived from a complex three-dimensional architecture with multiple branches resembling those of a tree [43]. These branches facilitate the adsorption of a greater number of analyte molecules and simultaneously generate abundant hot spots due to the combination of coupling in the spaces between adjacent branches and additive plasmonic effects, which increases their ability to enhance the local electric field [44,45].

Several techniques have been used to fabricate AgDs with different structural characteristics, such as chemical methods in aqueous media, ultraviolet irradiation of micelles with surfactants, and photoreduction under UV irradiation [46]. However, these methodologies have limitations, as they require specialized equipment, involve the use of hazardous materials, and necessitate the use of seed particles, templates, or multiple stabilizing agents, which increase production costs [41]. Electrochemical deposition (ED) is one of the most widely used techniques for synthesizing AgNSs, due to its simplicity, high degree of control, and scalability for large-scale production. ED methods include the application of constant current or voltage, voltage sweep cycles, and pulsed current or voltage cycles [47,48,49]. Among these, pulsed current electrodeposition (PCE) in a two-electrode configuration offers a robust and versatile strategy for controlling the nucleation and growth of particles under ambient conditions [50,51].

This work provides a novel contribution by systematically correlating galvanostatic anodization current density with the nucleation, growth, and final morphology of TiO_2_ nanostructures, and by linking these features to the formation of silver dendrites during pulsed electrodeposition. Unlike conventional potentiostatic anodization, this approach provides reproducible morphological control and enables the generation of vertically oriented dendritic architectures with high hot spot density. To the best of our knowledge, this is the first systematic study demonstrating how constant-current anodization can be exploited to tailor the oxide template for enhanced SERS performance, establishing a scalable and reproducible route for high-sensitivity substrates.

## 2. Materials and Methods

### 2.1. TNS Preparation

TiO_2_ structures were synthesized using titanium foil (grade II, 10 × 10 × 0.1 mm) as anode, positioned 1 cm apart from a graphite rod cathode (6 mm diameter, StonyLab, Nesconset, NY, USA). Prior to anodization, the titanium foils were cleaned in three sequential 10 min ultrasonic baths of acetone (C_3_H_6_O, Karal, México), ethanol (C_2_H_6_O, HYCEL, México), and deionized water. The electrodes were partially immersed in an organic electrolyte composed of 0.6 wt% ammonium fluoride (NH_4_F, Meyer, Vallejo, CA, USA), 2% deionized water, and monoethylene glycol (MEG, C_2_H_6_O_2_, Karal, México) under magnetic stirring. Constant current anodization was performed at 5–30 mA/cm^2^ for 30 min, with four independent replicates for each condition. The voltage response was monitored in situ using a UT117C Uni-T multimeter. Following anodization, the samples were rinsed with deionized water and ethanol, and annealed in air at 450 °C for 4 h.

### 2.2. TNS Decoration with AgNSs

A two-electrode setup was used for PCE of AgDs, employing the previously anodized TNS substrates (four independent replicates) as the cathode and a Pt sheet (15 × 15 mm × 0.1 mm, StonyLab, USA) as the anode. PCE was carried out in an aqueous solution containing 10 mM silver nitrate (AgNO_3_, Sigma-Aldrich, St. Louis, MO, USA) and 100 mM sodium nitrate (NaNO_3_, Meyer, USA) at room temperature, alternating 50 ms ON/250 ms OFF time. A custom-built Arduino-based system controlled the pulsed current deposition. The current density and the number of cycles were fixed at 5 mA/cm^2^ and 400, respectively. After deposition, all substrates were thoroughly rinsed with deionized water and dried in air.

### 2.3. Methylene Blue Solutions

A standard methylene blue dye (MB, C_16_H_18_ClN_3_S, HYCEL, México) was employed as the probe molecule to evaluate SERS performance. A solution with a concentration of 1 × 10^−11^ M was prepared using ethanol as solvent. Additionally, a 1 × 10^−4^ M solution was prepared to calculate the analytical enhancement factor (AEF). For all SERS measurements, a 10 μL aliquot of solution was deposited onto the substrates and dried under ambient conditions.

### 2.4. Raman Spectroscopy and Morphological Characterization

**Scheme 1:**
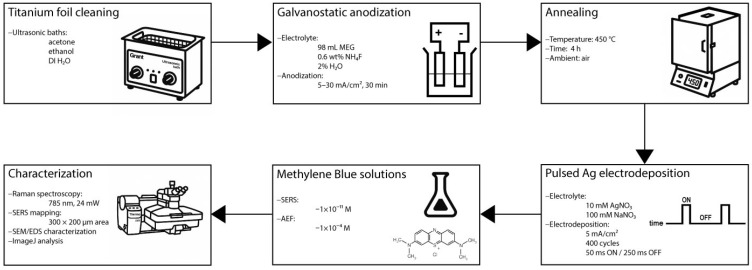
Flowchart summarizing the synthesis and characterization process described in this work.

Crystal phase identification and SERS measurements were performed using a confocal Raman microscope (Thermo Scientific DXR, Waltham, MA, USA) equipped with a 785 nm laser operating at 24 mW. All measurements were acquired at randomly selected locations across the sample surface, with a focused laser spot size of 50 μm. SERS mapping was conducted over a 300 × 200 μm area. Surface morphology was examined by field-emission scanning electron microscopy (FESEM, JEOL JSM-7600F, Peabody, MA, USA). SEM images were acquired using secondary electrons at 15 kV and a working distance of 5.0 mm. The tube length was measured from cross-sectional SEM images acquired in a JCM-6000 Benchtop SEM (Peabody, MA, USA). Tube diameters were determined using the ImageJ software (v1.53k), and average values were calculated from at least 30 measurements per sample. Porosity quantification was also performed using the same software. In samples where nanograss partially covered the surface (e.g., at 25 and 30 mA/cm^2^), tube diameters and porosity values were determined from regions where the tubular structure remained exposed. To provide a clear overview of the experimental procedure, the main steps of the synthesis and characterization are summarized in Scheme 1. The scheme illustrates the sequential process from titanium foil preparation to anodization, annealing, silver deposition, and final SERS evaluation.

## 3. Results

**Figure 1:**
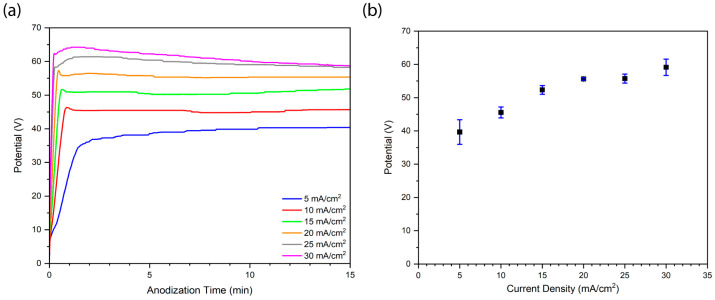
(**a**) Voltage–time profiles during galvanostatic anodization of Ti at different current densities (5–30 mA/cm^2^); (**b**) Steady-state potential as a function of current density.

The galvanostatic anodization mode, characterized by the application of a constant current density, allows the maintenance of a stable electric field and, consequently, an effective reaction rate. The current density (J) used in the anodization was varied between 5 and 30 mA/cm^2^. Figure 1a shows the potential (V) curves as a function of anodization time (min). In general terms, these curves exhibited an abrupt increase in potential in the first few seconds, followed by a moderate increase in the slope until reaching a maximum value, and finally stabilizing. This process is related to the initial growth of the metallic oxide layer, the sudden increase in potential due to the formation of pits in the barrier layer, and, finally, a gradual deceleration attributed to the development of TiO_2_ nanotubes [17,27]. Previous reports have demonstrated that, at J values higher than 1 mA/cm^2^, the edges of the nanotubes tend to collapse, which, over time, generates nanosponges and nanograss [10]. Figure 1b presents the average potential values recorded, as well as their standard deviation, for each J. For 5 mA/cm^2^, the average potential was 40 ± 4 V. In contrast, for 30 mA/cm^2^, the system established a potential of 60 ± 2 V.

**Figure 2:**
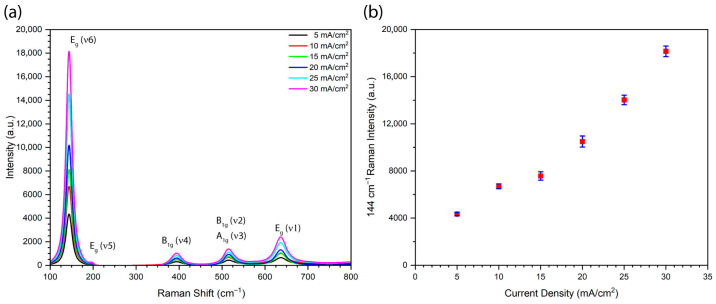
(**a**) Raman spectra of anodized TiO_2_ nanotube arrays obtained at different current densities (5–30 mA/cm^2^), showing the characteristic anatase modes. (**b**) Raman intensity of the E_g_ (v6) mode at 144 cm^−1^ as a function of current density.

**Figure 3:**
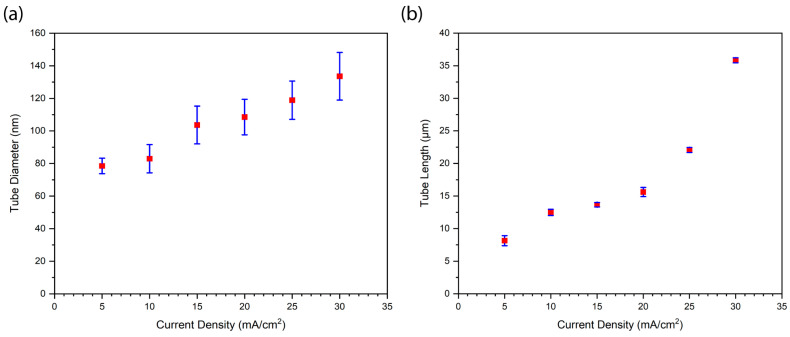
(**a**) Tube diameter and (**b**) length of the TiO_2_ nanostructures, measured for substrates anodized at current densities ranging from 5 to 30 mA/cm^2^.

Raman spectra of the fabricated substrates were obtained, as shown in Figure 2a, and in all cases corresponded to the anatase crystalline phase. The characteristic peaks of the TiO_2_ phase, E_g_ (ν6), E_g_ (ν5), B_1g_ (ν4), A_1g_ (ν3), B_1g_ (ν2), and E_g_ (ν1) were located at 144, 196, 394, 515, and 636 cm^−1^, respectively. It was observed that the signal intensity increased as the applied anodization current density increased, which is attributed to greater thickness and ordering of the oxide layer. This correlation indicates that current density acts as a control parameter, governing oxide morphology (tube diameter, length, and nanograss coverage), which in turn dictates the intrinsic Raman response. It is important to note that the increase in Raman intensity is not a direct effect of the applied current density, but rather a consequence of morphological changes in the oxide film [52,53]. As shown in Figure 3 and Table 1, higher current densities yielded thicker oxide layers and larger tube diameters, which enhance light–matter interaction and thus the Raman scattering efficiency of TiO_2_. The absence of additional peaks at ~447 and ~612 cm^−1^ confirms that no rutile domains are present, consistent with pure anatase formation. Previous studies established these Raman fingerprints as definitive markers of the anatase phase. These conditions favor a more intense Raman response. The uniformity of the samples was evaluated by analyzing the average intensity of the vibrational mode at 144 cm^−1^ in different regions of each substrate. Figure 2b presents the variation of this intensity as a function of the applied J. This variation showed an almost linear increase throughout the entire range of current densities evaluated. The error bars represent the standard deviation (SD) obtained from the measurements. The highest relative standard deviation was 5%, confirming good surface homogeneity in all the evaluated substrates.

**Figure 4:**
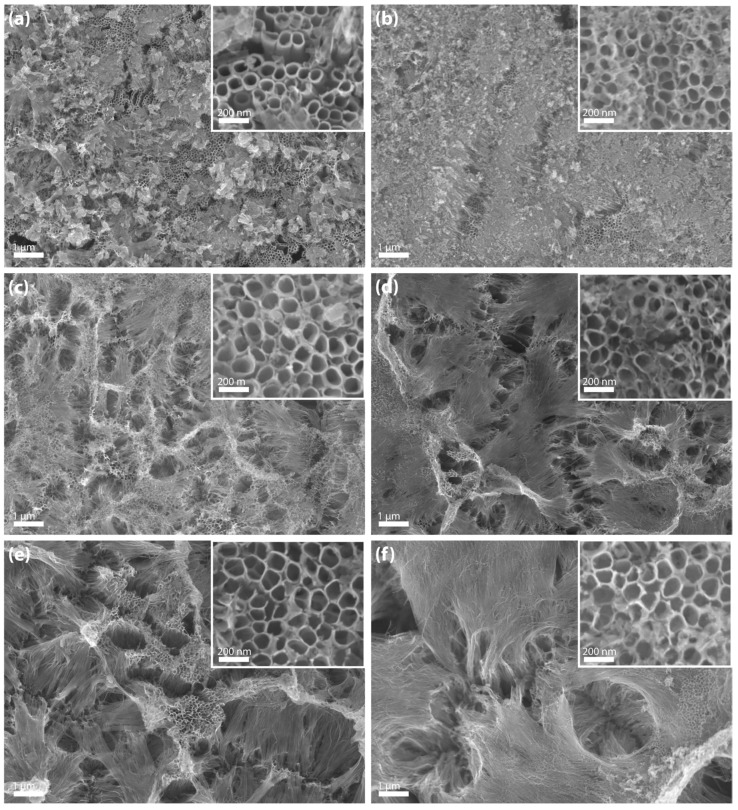
SEM images of TiO_2_ nanotube arrays anodized at different current densities: (**a**) 5, (**b**) 10, (**c**) 15, (**d**) 20, (**e**) 25, and (**f**) 30 mA/cm^2^. Insets show representative regions of exposed nanotubes from which tube diameters and porosity values were measured. Increasing the current density from 5 to 30 mA/cm^2^ results in an increase in tube diameter from 79 ± 5 nm to 134 ± 14 nm. Images were acquired at 10,000× magnification, with insets at 50,000×.

**Figure 5:**
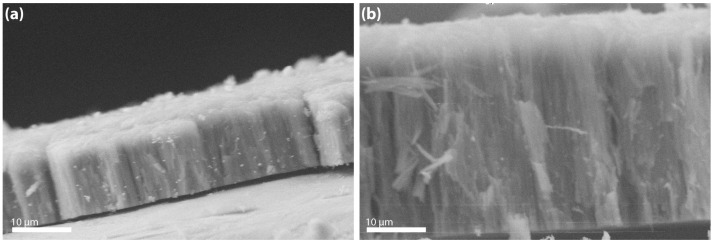
Cross-sectional SEM images of TiO_2_ nanotube arrays anodized at (**a**) 15 and (**b**) 30 mA/cm^2^. Images were acquired at 2000× magnification.

SEM images revealed the morphological evolution of the surfaces of the anodized TiO_2_ substrates across the evaluated current density range. Figure 3 shows the tube diameters (a) and the length (b) of the TiO_2_ nanostructures. At 5 and 10 mA/cm^2^ (Figure 4a,b), only scarce nanograss was observed, where the top of the nanotubes collapses and accumulates over the mouths of adjacent tubes. Additionally, nanotubes with an average diameter of 79 ± 5 nm and an estimated porosity of 25.9% were distinguished (Figure 4a,b). At 15 mA/cm^2^ (Figure 4c), nanograss becomes abundant but remains discontinuous, with locally exposed tube mouths still visible. At 20–25 mA/cm^2^ (Figure 4d,e), the nanograss layer was dense and continuous, whereas at 30 mA/cm^2^ (Figure 4f), the coverage is nearly total, leaving very few regions of the tubular structure visible; under this condition, an average diameter of 134 ± 15 nm was seen. The nanotube diameter strongly depends on the applied J [10], since under galvanostatic mode, the current controls the electric field and, consequently, the potential required to sustain it. The transition from nanotubes to nanograss arises from a dynamic balance between oxide growth and its chemical dissolution assisted by fluoride ions. Under high current density, not only is the rate of TiO_2_ formation increased, but the lateral dissolution of the tube walls is also intensified. This structural weakening, enhanced by deeper F^−^ penetration, leads to the loss of mechanical integrity of the tubes and their eventual collapse, thereby promoting nanograss formation [8]. The thickness of TiO_2_ was recorded in the range from 8 ± 1 µm to 36 ± 0.3 µm, within the evaluated current density interval. To further illustrate the vertical growth of the anodized nanostructures, cross-sectional SEM images of the samples anodized at 15 and 30 mA/cm^2^ are presented in Figure 5. These images confirm the progressive increase in oxide thickness with current density, ranging from ~13 µm at 15 mA/cm^2^ to over 35 µm at 30 mA/cm^2^, in agreement with the values summarized in Table 1.

The galvanostatic anodization regime provides direct control of the ionic flux through the oxide layer, as the imposed current density defines both the electric field and the dissolution/formation balance that drives nanotube nucleation. As reported by Zhang et al. [19], there exists a quantitative linear relationship between the anodizing current and the nanotube growth rate, with higher current densities leading to faster elongation of the nanotube arrays until a threshold beyond which chemical dissolution dominates. The initial stage corresponds to the formation of a compact oxide barrier, followed by pore nucleation through field-assisted dissolution in the presence of fluoride. Once established, the steady-state growth regime is characterized by a near-linear increase in tube length with the anodizing charge, a behavior typical of galvanostatic anodization. Moreover, Casado et al. [33] compared potentiostatic and galvanostatic anodization of titanium membranes and demonstrated that constant-current operation enhances the reproducibility of nanotube formation across porous supports, as it avoids the transient variations in current density inherent to the potentiostatic mode. These results underscore that the order and aspect ratio of nanotubes can be finely tuned by controlling the current density under galvanostatic conditions. Further mechanistic insights have been obtained by extending galvanostatic anodization to other valve metals. Wen et al. [54] reported the growth of tantalum oxide nanotubes under constant-current conditions. They observed that both tube length and diameter increased with current density up to an optimum window, while excessive current led to irregular morphologies due to over-etching. Similarly, Alijani et al. [32] demonstrated that high-aspect-ratio TiO_2_ nanotubes up to ~44 µm thickness could be achieved under galvanostatic anodization in lactic acid-modified electrolytes, with current density directly dictating the growth rate and structural robustness of the nanotube layers. Overall, the evidence indicates that low-to-moderate current densities favor controlled nucleation and uniform tube elongation, whereas excessively high currents accelerate chemical dissolution at the tube mouths, ultimately producing structural collapse and nanograss. In our results, the observed transition from ordered arrays at 5–15 mA/cm^2^ to partial disorder at 30 mA/cm^2^ fits consistently within this mechanistic framework. Thus, the galvanostatic approach enables the precise tailoring of tube morphology by directly adjusting the current density, which will later play a critical role in directing silver nucleation pathways during electrodeposition.

**Figure 6:**
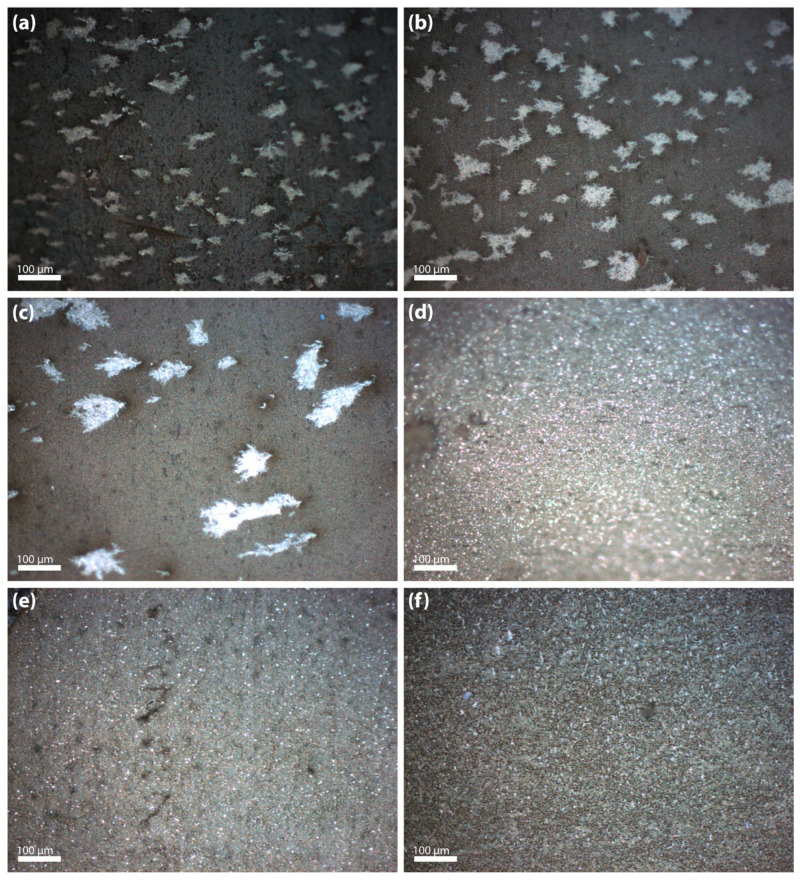
Optical microscopy images (10× objective) of TiO_2_ nanotube arrays after pulsed electrodeposition of silver at different anodization current densities: (**a**) 5, (**b**) 10, (**c**) 15, (**d**) 20, (**e**) 25, and (**f**) 30 mA/cm^2^.

Figure 6 shows the optical micrographs obtained with a 10× objective after the electrodeposition of silver nanostructures under identical conditions in all cases (5 mA/cm^2^, 400 cycles, 50 ms ON/250 ms OFF). In the samples with substrates anodized at 5–10 mA/cm^2^ (Figure 4a,b), only scarce nanograss was present. At 15 mA/cm^2^ (Figure 4c), nanograss was abundant but discontinuous, leaving locally exposed tube mouths; under these conditions, Ag nucleation remained localized and micrometer-scale dendrites formed (as seen in Figure 4). In contrast, the samples anodized at 20–30 mA/cm^2^ (Figure 6d–f) showed surfaces approaching quasi-full to nearly total nanograss coverage, favoring more homogeneous nanoparticle coatings.

**Figure 7:**
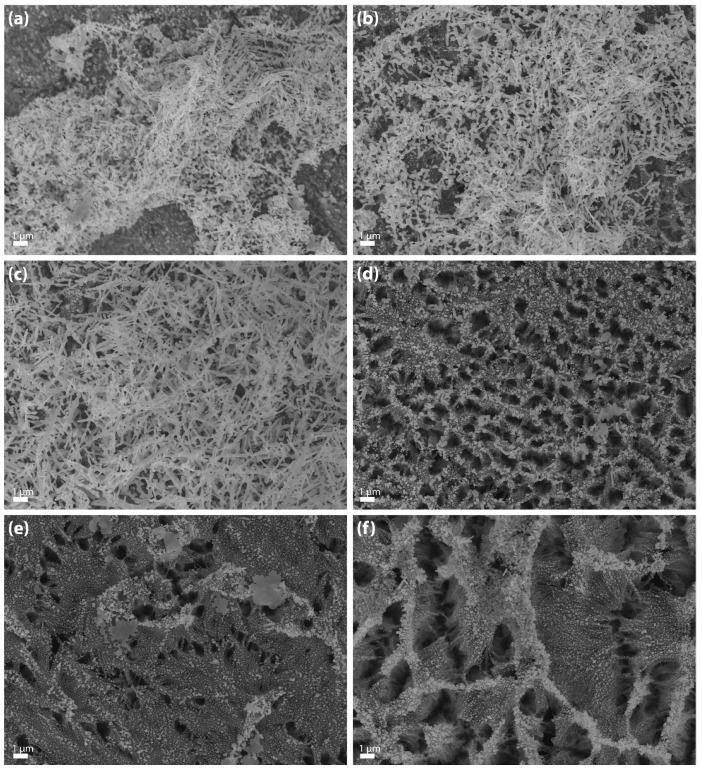
SEM micrographs of silver structures obtained by pulsed electrodeposition (5 mA/cm^2^, 400 cycles, 50 ms ON/250 ms OFF) on the anodized substrates at (**a**) 5, (**b**) 10, (**c**) 15, (**d**) 20, (**e**) 25, and (**f**) 30 mA/cm^2^. At lower current densities (**a**–**c**), discrete micrometer-scale dendrites with branched morphologies are observed, while at higher current densities (**d**–**f**), the deposition becomes more homogeneous and dominated by nanoparticle coverage. Images were acquired at 5000× magnification.

**Figure 8:**
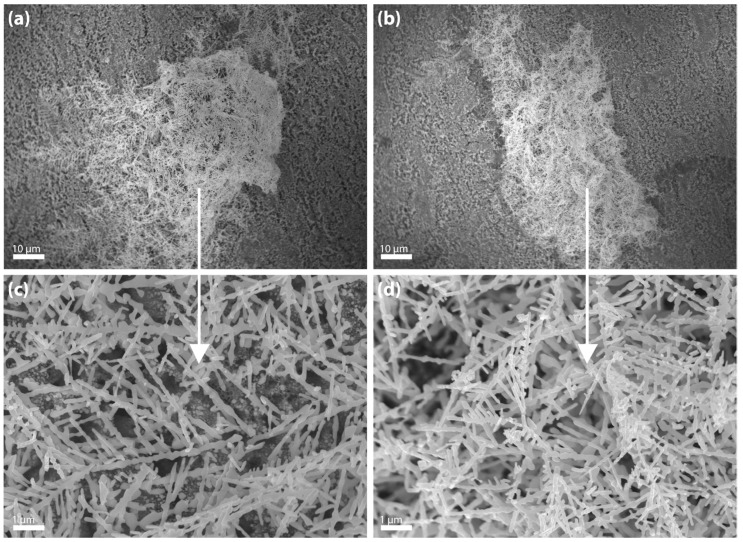
SEM images of two different dendrites in the sample anodized at 15 mA/cm^2^: (**a**) horizontally oriented dendrite, where the TiO_2_ substrate is visible in the background (**c**); (**b**) vertically oriented dendrite, showing the distinct vertical layers that compose it (**d**). Images were acquired at 1000× magnification, with close-ups at 10,000×.

Figure 7a–f show representative SEM micrographs of the structures formed after the PCE process of silver on the different anodized substrates previously characterized in Figure 4, Figure 5 and Figure 6. In all cases, the presence of AgNPs distributed across the entire surface was recorded; however, only specific substrates favored dendritic growth. These dendrites exhibited a well-defined branched morphology. For 5 mA/cm^2^ (Figure 7a), the dendrites reached an average size of 103 ± 16 μm in length, 43 ± 9 μm in width, and 5 ± 1 μm in height, while on the substrates anodized at 15 mA/cm^2^ (Figure 7c), the largest dendrites reached 206 ± 8 μm in length, 88 ± 26 μm in width, and 9 ± 2 μm in height. These structures preferentially grew on substrates with exposed nanotubes, as they serve as nucleation and preferential growth sites for such structures [55], generating a hierarchical coverage that combines the nanometric roughness of the particles with the microstructure of the dendritic branches (Figure 7a–c). It is well established that nanograss (Figure 7d–f) provides excellent adhesion for silver nanoparticles [56], which in turn may enhance the plasmonic response compared to nanotubes. However, dendrites—particularly those with 3D growth—have been reported to exhibit the best plasmonic performance, even enabling the detection of rhodamine 6G (R6G), 5,5’-dithio-bis-(2-nitrobenzoic acid) (DTNB), and Matrix Metalloproteinase (MMP-9) at concentrations of 1 × 10^−14^ [57], 1 × 10^−15^ [58], and 1 × 10^−17^ [59] M, respectively. The coexistence of these two morphological scales is highly relevant for SERS applications, as it promotes the formation of multiple hot spots at the junctions between nanoparticles and the intersections of dendritic branches, thereby optimizing Raman signal amplification. Figure 8 presents SEM images of two different dendrites (a,b) and their close-up (c,d,white arrow), where it is possible to see the AgNPs under the dendritic structure.

The mechanistic pathway for silver dendrite formation during pulsed electrodeposition can be rationalized through a sequence of electrochemical steps. In the aqueous electrolyte, silver nitrate dissociates completely:

$$AgNO_{3}\left(aq\right)\to Ag^{+}\left(aq\right)+NO_{3}^{-}(aq)$$

At the cathode (TiO_2_ nanotube substrate), the primary reduction involves the one-electron transfer of solvated silver ions:

$$Ag^{+}\left(aq\right)+e^{-}\to Ag^{0}(s)$$

Under diffusion-limited and high-field conditions, localized supersaturation at protrusions leads to rapid nucleation and growth. During the ON period of the pulse, the surface concentration of *Ag*^+^ decreases sharply, producing strong concentration gradients. This favors further reduction at high-curvature sites, where electric field crowding lowers the local energy barrier for deposition. In parallel, secondary reactions may occur, particularly at more negative potentials or in confined nanotube environments. Hydrogen evolution can proceed as:

$$2H_{2}O\left(l\right)+2e^{-}\to H_{2}\left(g\right)+2OH^{-}(aq)$$

This reaction locally increases pH near the cathode, altering ion solvation and modifying the growth kinetics of Ag nuclei. Moreover, the generated *OH*^−^ can interact with *Ag*^+^ to form transient silver hydroxide:

$$Ag^{+}\left(aq\right)+OH^{-}\left(aq\right)\to AgOH(s)$$

which subsequently dehydrates under cathodic bias to metallic silver:

$$2AgOH\left(s\right)\to Ag_{2}O\left(s\right)+H_{2}O\left(l\right)$$

$$Ag_{2}O\left(s\right)+H_{2}O\left(l\right)+2e^{-}\to 2Ag\left(s\right)+2OH^{-}(aq)$$

At the platinum anode, the complementary process is oxygen evolution:

$$2H_{2}O\left(l\right)\to O_{2}\left(g\right)+4H^{+}\left(aq\right)+4e^{-}$$

Thus, the overall cell reaction during silver electrodeposition can be expressed as:

$$4Ag^{+}\left(aq\right)+2H_{2}O\left(l\right)\to 4Ag\left(s\right)+O_{2}+4H^{+}(aq)$$

The pulsed waveform modulates these reactions dynamically. During OFF periods, *Ag*^+^ replenishment from the bulk re-establishes local supersaturation, while *H*_2_ bubbles partially desorb, preventing surface passivation. During ON periods, over-limiting current drives the reduction of both free *Ag*^+^ and intermediate *Ag*–*OH* species, sustaining anisotropic branching. This cyclic interplay of ion transport, nucleation, and competitive side reactions explains why the system favors fractal dendritic architectures rather than compact films, consistent with classical models of electrocrystallization under mass-transport control [60,61,62,63,64]. These electrochemical steps define the chemical environment under which silver growth proceeds; however, the ultimate dendritic architecture is dictated by the interplay of these reactions with the electric field distribution and the TiO_2_ nanotube geometry, as discussed below.

Complementary to the electrochemical reactions that govern *Ag*^+^ reduction and side processes, the architecture of the TNS nanotubes further biases Ag growth toward tip-dominated, anisotropic branching during pulsed electrodeposition. Field crowding at tube mouths and edges concentrates the local current density; once initial Ag nuclei form, the enhanced electric field at protruding tips accelerates ion reduction and sustains fractal branching rather than isotropic film thickening. Classic and modern electrocrystallization studies show that edge/tip fields and adsorbate transport govern the particle-to-dendrite transition and stabilize growth at tips, consistent with our observed ramified morphologies [36,60]. Under pulsed deposition, ON periods drive rapid *Ag*^+^ depletion at active tips, while OFF periods allow partial diffusion-layer recovery, preventing surface passivation and renewing supersaturation at high-field sites; in silver systems, this pulse-controlled mass transport reliably promotes dendritic textures versus compact films [61,65]. When the instantaneous current exceeds the diffusion-limited value, over-limiting regimes emerge inside nanoscale pores/cavities. In such confinement, surface conduction and space-charge effects direct cation flux along walls toward protrusions, reinforcing dendritic advance and branch multiplication—mechanisms verified in nanoporous templates and directly linked to controllable dendrite growth [62]. These branching pathways have direct consequences for SERS. As already discussed in Figure 7 and Figure 8, this dendritic growth mechanism directly impacts the SERS signal, since the 3D branched architecture ensures a dense and spatially distributed network of hot spots. This interpretation is consistent with previous demonstrations showing that 3D Ag dendrites outperform planar nanoparticle films by providing hierarchical plasmonic coupling and multi-resonant responses [45,66,67].

This three-dimensional (3D) arrangement allows the excitation light to couple more effectively with multiple plasmonic junctions along dendritic branches, enhancing the overall effective field intensity. Comparative studies have confirmed that while planar nanoparticle assemblies can generate localized but intense electromagnetic fields, dendritic and fractal-like architectures create extended networks of coupled plasmonic hot spots, resulting in more reproducible and intensified SERS signals (e.g., nanopillars, dendritic structures, and porous foams exhibit denser, isotropic hot spot distributions than 2D substrates) [66]. Additionally, dendritic Ag nanostructures display significantly higher SERS performance compared to Ag nanoparticle films, a direct consequence of their vast surface area and densely packed hot spots in nanogaps between multilayered branches [46].

Building on this mechanistic picture, the ON/OFF waveform and the total number of cycles can be tuned to steer branch density and multiscale roughness without changing the total charge delivered. During pulsed electrodeposition on TiO_2_ nanotubes, the ON/OFF waveform can be used to decouple charge delivery from mass transport and thus steer hierarchy. Shorter T_on_ and lower duty cycles enhance diffusion-layer recovery during T_off_, raising the effective nucleation rate and favoring finer primary branches and higher branch density; conversely, longer T_on_ (higher duty) promotes growth thickening and coalescence. This pulse–transport balance—where frequency and duty cycle modulate local supersaturation and instantaneous limiting current at protrusions—is a well-established outcome of pulse plating theory and experiments [68]. For silver specifically, studies show that the pulse profile (duty, frequency, and peak current) governs the transition from particle-like deposits to tip-dominated, anisotropic dendrites, consistent with field crowding at edges and periodic renewal of ionic supply [61]. In confined architectures (nanotubes/porous scaffolds), operating near the diffusion-limited regime enhances anisotropic advance: under over-limiting conditions, surface conduction and space-charge in nanopores funnel cations toward protrusions, reinforcing branch multiplication across pulses and enabling 3D frameworks rather than uniform films [62]. Within such dendritic networks, hierarchical roughness and interbranch nanogaps create volumetric EM hot spot distributions; SERS studies on 3D Ag dendrites consistently attribute significant gains to plasmonic coupling across multiscale branches, while also noting that excessive growth closes gaps and reduces enhancement—hence the practical need to cap the cycle number at the point where hierarchy is maximized but coarsening has not yet occurred [45,69].

**Figure 9:**
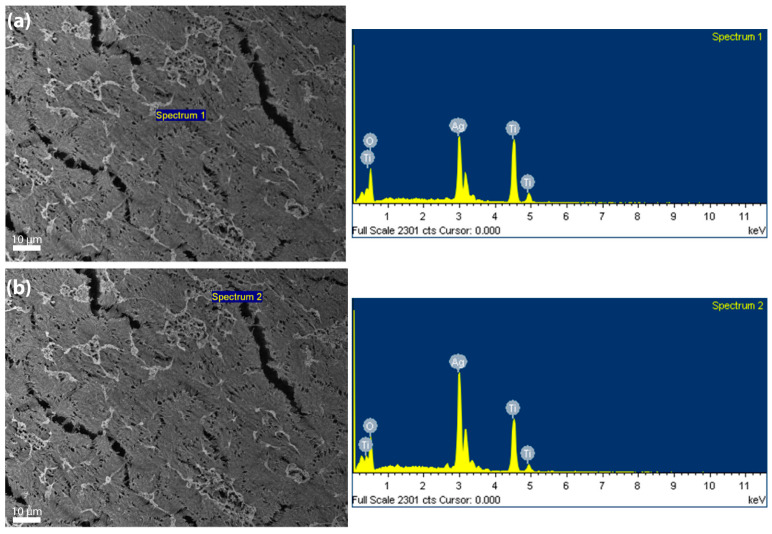
SEM–EDS of the sample anodized at 25 mA/cm^2^. (**a**) SEM top view with Spectrum 1 acquisition point (**left**) and the corresponding EDS spectrum (**right**). (**b**) SEM top view with Spectrum 2 acquisition point (**left**) and the corresponding EDS spectrum (**right**).

Figure 9 shows two representative point spectra acquired on the sample anodized at 25 mA/cm^2^ and subsequently subjected to pulsed electrodeposition. In both regions (Spectrum 1–2), the spectra present pronounced Ag lines (~3.0–3.2 keV) together with Ti (~4.5–4.9 keV) and O (~0.53 keV) signals from the underlying TiO_2_. The higher Ag peak intensity in panel (b) is consistent with the thicker/denser Ag coverage observed locally in SEM. These results confirm the presence and spatial localization of Ag on the anodized TiO_2_ scaffold.

Methylene blue (MB) is one of the most widely used probe molecules for evaluating the performance of SERS substrates, since it exhibits well-established and easily identifiable vibrational modes, allowing precise comparison under different experimental conditions. Table 2 summarizes the main vibrational modes (cm^−1^) of MB reported in the literature [35,70,71]. In this study, the 1610 cm^−1^ vibrational mode was selected as the primary reference for the SERS mappings. This mode corresponds to C–C ring stretching vibrations and is particularly sensitive to the local electromagnetic environment, which explains its widespread use as a robust SERS reporter peak. Other prominent modes include the symmetric C–N stretching at 1396 cm^−1^ and the asymmetric C–N/C–C coupled vibrations around 1430–1442 cm^−1^, which provide complementary markers of molecular adsorption and orientation on plasmonic nanostructures [71]. Meanwhile, the in-plane C–H bending bands (833–1154 cm^−1^) are weaker in intensity but remain helpful in confirming molecular identity, and the C–N stretching region at 1180–1226 cm^−1^ is highly sensitive to charge transfer effects between MB and metallic substrates [72]. Taken together, these vibrational features provide a comprehensive fingerprint of MB, allowing correlations between peak intensity and hot spot distribution across different morphologies.

**Figure 10:**
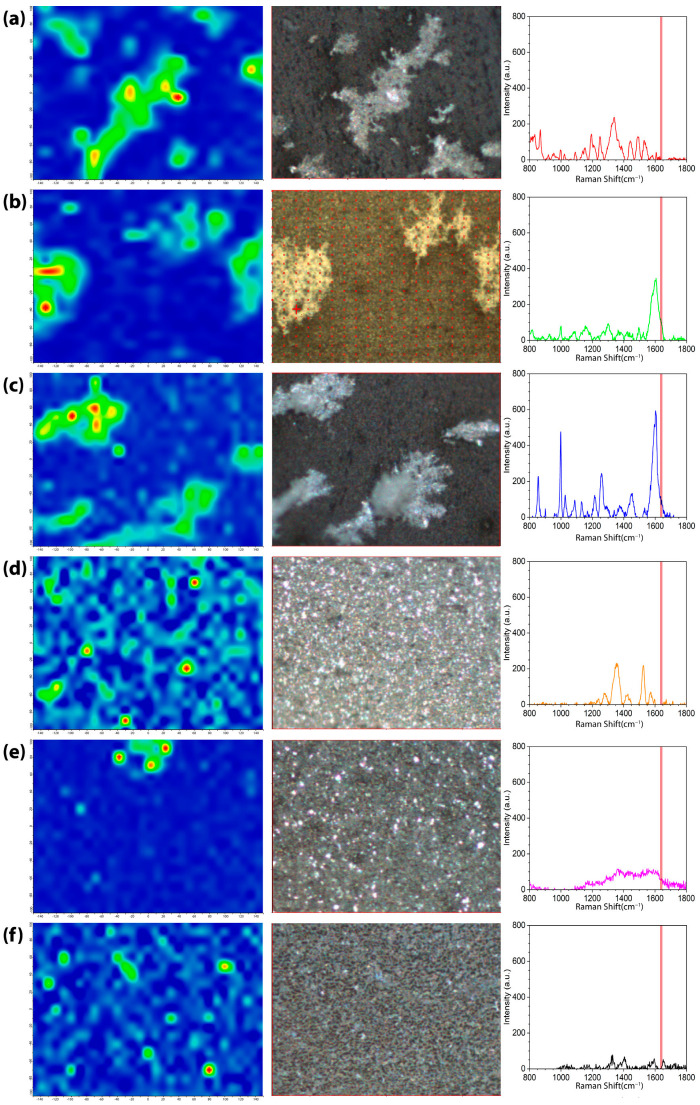
SERS intensity maps at 1610 cm^−1^ (**left column**), corresponding optical micrographs (**center column**), and representative spectra (**right column**) for MB adsorbed on Ag-modified TiO_2_ substrates obtained by pulsed electrodeposition at different current densities: (**a**) 5, (**b**) 10, (**c**) 15, (**d**) 20, (**e**) 25, and (**f**) 30 mA/cm^2^. The red line indicates the 1610 cm^−1^ Raman mode as a reference. Color in the intensity maps (left column) indicates relative signal (a.u.; warm = higher, cool = lower).

Figure 10a–f show, for each anodized substrate coated with silver, the SERS intensity mapping corresponding to the 1610 cm^−1^ mode (left column), the optical micrograph of the mapped area (central column), and a representative SERS spectrum of the red regions in the mapping (right column). These results reveal a clear correlation between the initial TiO_2_ morphology, the type of silver structure formed by electrodeposition, and the distribution of hot spots. At 5 mA/cm^2^ (Figure 10a), the SERS signals were mainly concentrated in regions with dendritic aggregates, generating isolated points of high intensity (red areas in the mapping). Similarly, at 10 mA/cm^2^ (Figure 10b), the hot spots were distributed around the dendritic formations, confirming their role as dominant active sites. At 15 mA/cm^2^ (Figure 10c), the most intense SERS response was recorded, with a high density of hot spots [72,73,74]. In contrast, at 20 and 25 mA/cm^2^ (Figure 10d,e), the mappings exhibited lower overall intensity and fewer hot spots. Finally, at 30 mA/cm^2^ (Figure 10f), the SERS signal was very weak, consistent with a uniform and continuous 2D coating of silver nanoparticles on the nanograss, which drastically reduced hot spot generation.

The analytical enhancement factor (*AEF*) was determined using Equation (1) [75] and from the values in Table 3, which relates the SERS signal intensity *(I_SERS_*) and analyte concentration (*C_SERS_*) on the active substrate to the corresponding values measured on a non-enhancing reference (*I_ref_* and *C_ref_*):

(1) $$AEF=\left(\frac{C_{ref}}{C_{SERS}}\right)\left(\frac{I_{SERS}}{I_{ref}}\right)$$

The *AEF* value was calculated for the characteristic Raman mode 1610 cm^−1^ of MB, using a 1 × 10^−4^ M solution of MB as the reference. Notably, the value reached falls within the upper range reported for functionalized AgNP-TiO_2_ Nanotube substrates [74]. Although higher AEFs (>10^7^) have been demonstrated using advanced architectures or resonance-enhanced SERS, the magnitude obtained here underscores the efficiency of the electrodeposition protocol.

To contextualize the performance of this study, Table 4 presents a comparative summary of recent SERS substrates based on Ag-decorated TiO_2_ nanostructures for the detection of Methylene Blue and other analytes. Various fabrication methods are represented, including DC magnetron sputtering, thermal evaporation, sol–gel synthesis, and ultraviolet-induced deposition. While these approaches can achieve competitive enhancement factors, they typically rely on vacuum-based or multi-step chemical processes, which limit scalability and increase fabrication complexity.

It should be emphasized that there are very few studies employing Ag nanoparticles or silver dendrites directly grown on TiO_2_ nanostructures obtained by anodization for SERS applications. Most anodized TiO_2_/Ag systems reported in the literature have been explored mainly for photocatalysis, dye degradation, or lithium-ion batteries rather than SERS applications. For instance, Ag-doped TiO_2_ is extensively used in photocatalytic degradation of organic dyes and pollutants [76], and anodized TiO_2_ nanotubes are primarily developed as photocatalysts or anodes for lithium-ion batteries [77]. In some cases, TiO_2_ nanotubes coated with Ag by sol–gel or chemical routes have shown improved catalytic activity [78,79], but their application in SERS remains scarce. To our knowledge, no previous study has combined galvanostatic anodization of TiO_2_ with pulsed Ag electrodeposition for SERS substrates, making our work the first of its kind.

Compared to potentiostatic anodization routes combined with sputtering or chemical reduction, our approach achieves competitive sensitivity (MB detection at 1 × 10^−11^ M with an AEF of 7 × 10^7^) through a simple and reproducible process. The ability to obtain highly sensitive SERS substrates using constant current anodization followed by electrodeposition, without relying on vacuum systems or stabilizing agents, highlights not only the novelty of this method but also its potential for scalability in practical SERS applications. It is also worth noting that the most comparable study available in the literature [80], which employed constant current electrodeposition for Ag dendrites combined with constant voltage anodization for TiO_2_ nanotubes, only reported SERS detection down to 1 × 10^−5^ M. This further emphasizes the advantage of the present approach, where constant current anodization is demonstrated for the first time as a more effective and reproducible route to achieve ultrasensitive TiO_2_/Ag SERS substrates.

In addition to sensitivity, two further aspects are essential for practical SERS applications: scalability and stability. The fabrication route presented here—galvanostatic anodization combined with pulsed Ag electrodeposition—offers intrinsic scalability, as both processes are low-cost, solution-based, and adaptable to large-area substrates without the need for vacuum systems [81,82]. This contrasts with sputtering and evaporation techniques, where high infrastructure cost and limited throughput restrict practical deployment. Regarding stability, it is well established that silver nanostructures, particularly dendritic morphologies, are prone to oxidation and surface restructuring, which can affect signal reproducibility over time. Strategies such as protective coatings or alloying (e.g., Ag@Au shells) have been shown to enhance stability while preserving plasmonic activity significantly [83,84]. Although long-term stability tests were not performed here, our results demonstrate a scalable and highly sensitive platform, while highlighting durability as a key direction for future optimization toward real-world sensing.

**Table 4:** Comparative performance of Ag-decorated TiO_2_ nanostructured SERS substrates.

Substrate Composition	Synthesis Method	Analyte	Analytical Enhancement Factor	Lowest Concentration Detected (M)	Ref.
AgNDs/ TiO_2_ Nanotubes	Constant Current Electrodeposition/ Constant Voltage Anodization	MB	--	1 × 10^−5^	[80]
AgNPs/ TiO_2_ Nanotubes	Thermal Evaporation/ Constant Voltage Anodization	MB	2 × 10^5^	1 × 10^−8^	[85]
Ag@mTiO_2_ NPs	Hydrothermal process/ Chemical Method	MB	--	1 × 10^−8^	[86]
AgNPs/ TiO_2_	Ultraviolet Light-Induced/ Sol–Gel	R6G	--	1 × 10^−8^	[87]
AgNPs/ TiO_2_ Nanotubes	DC Magnetron Sputtering/ Arc Ion Plating Method	R6G	1 × 10^9^	1 × 10^−8^	[88]
AgNPs/ TiO_2_ Nanosheet	DC Magnetron Sputtering/ Hydrothermal Method	R6G	1 × 10^5^	1 × 10^−9^	[89]
AgTNP@TiO_2_@Ag core-satellite	Chemistry Method	MB	--	1 × 10^−10^	[90]
AgDs/ TNS	Pulse Current Electrodeposition/ Constant Current Anodization	MB	7 × 10^7^	1 ×10^−11^	this work
AgNPs/ TiO_2_ Nanotubes and Nanograss	DC Magnetron Sputtering/ Constant Voltage Anodization	MB	1 × 10^5^	1 × 10^−12^	[74]
AgNPs/ TiO_2_ Nanorods	Chemistry Method	MG	4 × 10^5^	1 × 10^−12^	[91]
AgNPs/ TiO_2_ nanospheres	Ultraviolet Light-Induced/ Commercial Nanospheres	R6G	7 × 10^10^	1 × 10^−12^	[92]
AgNPs/ TiO_2_ Nanotubes	DC Magnetron Sputtering/ Constant Voltage Anodization	R6G	--	1 × 10^−14^	[93]

R6G: Rhodamine 6G; MB: Methylene Blue; MG: Malachite Green.

While methylene blue is one of the most widely used probe molecules for benchmarking SERS substrates due to its strong and well-defined vibrational modes, it should be noted that its structural and adsorption properties may not represent all possible analytes. MB is a small, cationic, and aromatic dye that interacts favorably with plasmonic surfaces, which facilitates reliable signal generation. However, analytes with different molecular weights, charges, or functional groups—such as large proteins or negatively charged biomolecules—may exhibit distinct adsorption behaviors and lower enhancement factors. This limitation has been previously acknowledged in SERS literature, where probe molecule properties can strongly influence enhancement mechanisms and adsorption affinities [34]. Thus, MB provides a robust and reproducible reference for evaluating the intrinsic performance of our Ag/TiO_2_ substrates. However, additional validation with molecules of different chemical structures and adsorption characteristics would be necessary to extend the applicability of the system to more complex analytes.

Our analysis of silver nucleation and dendritic growth was based on morphological evolution and its correlation with SERS performance. According to the literature, the surface chemistry of anodic TiO_2_ is also an important factor: oxygen vacancies have been identified as preferential anchoring sites for Ag nucleation [94], while hydroxylated surfaces promote stronger interactions with solvated species [95]. Such effects complement the structural aspects emphasized in our study, and they are consistent with reports that both morphology and surface chemistry co-govern nucleation behavior and SERS reproducibility [96]. By focusing on morphology, our results highlight the structural pathway for optimizing hotspot density, while remaining aligned with the broader understanding that chemical and structural factors interact to determine SERS performance.

## 4. Conclusions

The current density applied during galvanostatic anodization acts as a control parameter that defines the morphology of TiO_2_ and, consequently, the architecture of the silver structures generated by pulsed electrodeposition. In this context, the enhancement of the Raman signal is not a direct effect of the current density itself, but of the morphological changes it induces, including tube diameter, oxide thickness, and nanograss coverage. It was found that 15 mA/cm^2^ represents the optimal condition for obtaining 3D dendrites capable of producing a dense and homogeneous distribution of vertically oriented hot spots, which translates into a significantly enhanced SERS signal and enables the detection of analytes, such as methylene blue, at concentrations as low as 1×10^−11^ M. Moreover, the optimized substrate exhibited an analytical enhancement factor of 7 × 10^7^, confirming its strong amplification capability. This positions our substrate as a promising candidate for various SERS applications, with a performance comparable to that reported in the literature. Overall, these findings confirm that the precise control of oxide morphology prior to metal deposition is an effective strategy for developing high-performance SERS substrates.

## Figures and Tables

**Table 1:** Measured parameters of TiO_2_ anodized substrates at current densities ranging from 5 to 30 mA/cm^2^.

Current Density (mA/cm^2^)	Potential (V)	144 cm^−1^ Mode Intensity (a.u.)	Tube Diameter (nm)	Surface Porosity (% ± 1)	Tube Length (µm)
5	40 ± 4	4340 ± 132	79 ± 5	26	8 ± 1
10	46 ± 2	6697 ± 204	83 ± 9	29	13 ± 1
15	52 ± 1	7573 ± 359	104 ± 12	33	14 ± 1
20	55 ± 1	10,491 ± 469	109 ± 11	38	16 ± 1
25	56 ± 1	14,026 ± 399	119 ± 12	-	22 ± 1
30	60 ± 2	18,150 ± 447	134 ± 14	-	36 ± 1

**Table 2:** Assignments of major peaks for methylene blue (MB) [35,70,71].

SERS (cm^−1^)	Band Assignments
833, 885–897	In-plane bending of C–H
949–951	In-plane bending of C–H
1036–1070	In-plane bending of C–H
1119–1122	In-plane bending of C–H
1154	In-plane bending of C–H
1180–1185	Stretching of C–N
1205–1226	Stretching of C–N
1265	In-plane ring deformation of C–H
1301–1326	In-plane ring deformation of C–H
1396	Symmetrical stretching of C–N
1430–1442	Asymmetrical stretching of C–N
1501–1563	Asymmetrical stretching of C–C
1592–1597	Ring stretching of C–C
1601–1618	Ring stretching of C–C

**Table 3:** Raman intensities of the reference substrate without AgNPs (NO-SERS) at 1 × 10^−4^ M and of the Ag-decorated TiO_2_ SERS substrates at 1 × 10^−11^ M.

Concentration (M)	1610 cm^−1^ Raman Intensity (a.u.)
Ref (NO-SERS)	87
SERS 1 × 10^−11^	596

## Data Availability

The original contributions presented in this study are included in the article. Further inquiries can be directed to the corresponding authors.
